# Proteomic analysis of injured storage roots in cassava (*Manihot esculenta* Crantz) under postharvest physiological deterioration

**DOI:** 10.1371/journal.pone.0174238

**Published:** 2017-03-24

**Authors:** Yuling Qin, Astride Stéphanie Mouafi Djabou, Feifei An, Kaimian Li, Zhaogui Li, Long Yang, Xiaojing Wang, Songbi Chen

**Affiliations:** 1 Guangdong Provincial Key Laboratory of Biotechnology for Plant Development, College of Life Sciences, South China Normal University, Guangzhou, China; 2 Tropical Crops Genetic Resources Institute Chinese Academy of Tropical Agricultural Sciences/Key Laboratory of Ministry of Agriculture for Germplasm Resources Conservation and Utilization of Cassava, Danzhou, China; 3 Laboratory of plant physiology, Department of Biological Science, Higher Teachers´ Training College, University of Yaounde I, Yaounde, Cameroon; 4 Agricultural Bureau of Wuming County, Wuming, China; 5 Subtropical Crops Research Institute, Guizhou Provincial Academy of Agricultural Sciences, Xingyi, China; Shanghai Institutes for Biological Sciences, CHINA

## Abstract

Postharvest physiological deterioration (PPD) is a global challenge in the improvement of cassava value chain. However, how to reduce cassava spoilage and reveal the mechanism of injured cassava storage roots in response to PPD were poorly understood. In the present study, we investigated the activities of antioxidant enzymes of cassava injured storage roots in PPD-susceptible (SC9) and PPD-tolerant (QZ1) genotypes at the time-points from 0h to 120h, and further analyzed their proteomic changes using two-dimensional electrophoresis (2-DE) in combination with MALDI-TOF-MS/MS. Ninety-nine differentially expressed proteins were identified from SC9 and QZ1 genotypes in the pairwise comparison of 24h/0h, 48h/0h, 72h/0h and 96h/0h. Of those proteins were associated with 13 biological functions, in which carbohydrate and energy metabolism related proteins were the biggest amount differential proteins in both genotypes, followed by chaperones, DNA and RNA metabolism, and defense system. We speculated that SOD in combination with CAT activities would be the first line of defense against PPD to support PPD-tolerant cassava varieties. The four hub proteins including CPN60B, LOS2, HSC70-1 and CPN20B, produced from the network of protein-protein interaction, will be the candidate key proteins linked with PPD. This study provides a new clue to improve cassava PPD-tolerant varieties and would be helpful to much better understand the molecular mechanism of PPD of cassava injured storage roots.

## Introduction

Cassava (*Manihot esculenta* Crantz) is a staple food crop in Africa, Latin America and Asia [1, 2]. However, the rapid post-harvest physiological deterioration (PPD), a unique phenomenon in harvesting storage roots, has become a major constraint for extending cassava shelf-life compared with other root crops [3]. PPD is an active process involving in the changes of gene expression, novel protein synthesis and secondary metabolite accumulation [4–6]. Undesirable vascular streaking developed quickly was observed in the harvest storage roots and then caused deterioration within 2-3d. Because of PPD phenomenon, cassava storage roots have to be consumed soon after harvest [7].

The previous studies have been carried out to understand the biochemical and molecular mechanism of PPD [8–10]. PPD in cassava storage roots has been shown to be associated with an oxidative burst [11–16]. Iyer *et al*. (2010) showed that superoxide dismutase (SOD), catalase (CAT), and peroxidase (POD) in storage roots were more highly expressed in the regions closer to the site injured by machine [17]. This oxidative burst was reported to associate with cyanide production, which is rapid response to the mechanical injured in cassava storage roots [18]. Overexpressing CAT and SOD genes in cassava storage roots could availably decrease the accumulation of reactive oxygen species (ROS) independently from the ascorbate pool, and then reduce the PPD onset [19].

PPD is a major challenge for increasing cassava value chain. This phenomenon significantly reduces shelf-life of cassava storage roots for fresh consumption and decreases the income of smallholders. The previous reports indicated that extending the shelf-life of cassava to several weeks would reduce financial losses by $2.9 billion in Nigeria alone over a 20 years period [20]. Many scientists have made a lot of efforts to prevent or reduce the occurrence of PPD. Sánchez *et al*. (2006) reported that yellow-root cassava cultivars with higher β-carotene content have a delayed onset of PPD by 1 to 2 d [21]. In addition, Morante *et al*. (2010) surveyed different cassava germplasm resources and found three genotypes with high total carotenoid contents could delay PPD for up to 40 d after cassava harvesting [22], suggesting carotenoid may act as the roles of antioxidants. Pruning cassava plant could delays PPD before harvesting for a few days, but it reduced the dry matter content of the storage root [7].

Initial studies of PPD were focused on the changes of gene expression [14]. However, it was difficult to understand the PPD trigger mechanism and global regulation without the assistance of proteomic dataset. With the release and annotation of the cassava genome [23–25], it is possible to expand cassava proteome coverage and better characterize its modulation during PPD. Owiti *et al*. (2011) used the isobaric tags for relative and absolute quantification (iTRAQ) to analyze the proteomic changes during the early and late PPD. A total of 2600 proteins in cassava storage root were identified. Their functions included ROS scavenging, programmed cell death, defense response, signaling and cell-wall metabolism [26]. Vanderschuren *et al*. (2014) reported that about 300 proteins with significant abundance regulation during PPD were identified. They involved in oxidative stress, phenylpropanoid biosynthesis (including scopoletin), glutathione cycle, fatty acid α-oxidation, folate transformation and reduction II of sulfate. Among the identified proteins, the glutathione peroxidase (GPX) used glutathione to detoxify hydrogen peroxide (H_2_O_2_) and was considered a candidate for reducing PPD. Over-expressing GPX in *Arabidopsis thaliana* showed that PPD delay was probably associated with the reduction of lipid peroxidation and H_2_O_2_ accumulation [27]. Those results will provide a strategy to delay PPD in cassava storage roots through reducing ethene biosynthesis and increasing enzymes involved in suberization and lignifications. Ansari *et al*. (2014) reported that a burst of endogenous ethylene under stress revealed the fruits stayed at a high risk of pathogen infection, and caused various physiological disorders, then became deteriorations [28].

Through the previous studies had showed the differences linked with PPD in cassava as described above, proteomic changes of cassava injured storage roots in response to PPD were poorly reported. In the present study, we measured H_2_O_2_ and antioxidant activities in injured storage roots under PPD between PPD-susceptive (SC9) and PPD-tolerant (QZ1) genotypes stored at room temperature for 120h. In order to further solve cassava spoilage from understanding PPD molecular mechanism, we used comparative proteomics to explore the globally differential proteins and construct their interaction to speculate their potential relationship associated with PPD. These results will give insights into the molecular mechanism in response to PPD for the improvement of cassava breeding.

## Materials and methods

### Plant material preparation

Cassava genotypes QZ1 and SC9 were planted at Cassava Germplasm Bank of Tropical Crops Genetic Resources Institute, Chinese Academy of Tropical Agricultural Sciences (CATAS-Danzhou campus, China), and carefully harvested after 10 months. The halved storage roots with uniform size were randomly stored at 26°C to 28°C and 70% to 80% relative humidity. After 0, 24, 48, 72, 96, and 120 h, the injured storage roots were taken out and frozen in liquid nitrogen subsequently, and then stored at -80°C for use. Three storage roots, taken from three cassava plants, respectively, were used as one replicate, and three biological replicates were conducted in the present study.

### Determination of dry matter content

Cassava storage roots were carefully harvested, and cut into pieces. The dry matter content, expressed as the percentage of dry weight relative to fresh weight, was determined according to the method GB/T12087-2008 [29].

### Measurement of β-carotene content using high performance liquid chromatography (HPLC)

β-carotene content was measured using HPLC according to the method described from Yang et al. (2015) [30]. Cassava storage root was ground to a fine powder using the liquid nitrogen with mortar and pestle, and 1.0 g of sample powder was fully mixed with 2 ml cooled acetone extraction, and then added 2 ml cooled petroleum ether, centrifuged at 3000 rpm for 5 min at 4°C. The suspension was transferred to a new tube, and 1 ml of cooled petroleum ether was added to the residue. This procedure was repeated three times until the residue became colorless. The all suspensions were dried using nitrogen using Termovap Sample Concentrator. The dried extract was re-dissolved in 500 ml acetone, and collected the dissolved fraction using a disposable syringe. After filtered, the dissolved fractions were collected to a brown bottle for HPLC (Agilent 1260 Infinity LC) analysis. Samples were separated on a Waters YCM Carotenoids S-3 column (4.6 mm × 250 mm), and isocratic eluted with solvent system (methanol: tert-butyl methyl ether = 7:3). The samples were read at a wavelength of 450 nm. β-carotene content was calculated using the following formula: β-carotene contents (μg/g) = Ax ×Cs (μg/ml) × V (ml)/As × W (g); where Ax is the peak area of sample β-carotene absorbance, Cs (μg/ml) is the concentration of standard sample, V (ml) is the total extract volume, As is the area of standard absorbance, W (g) is the sample weight.

### Visual PPD evaluation

PPD evaluation of cassava storage roots stored at incubated rooms at 0, 24, 48, 72, 96, and 120 h was conducted. Quantitation of vascular discoloration was done on captured images using Image J software (http://rsb.info.nih.gov/ij/, NIH, MD, USA) [19].

### Determination of H_2_O_2_ content and antioxidant capacity

The content of H_2_O_2_ and the activities of SOD, CAT, and POD were determined using commercial assay kits (Nanjing Jiancheng Bioengineering Institute, Jiangsu, China) according to the manufacturer’s instruction Hu *et al*. (2016) [31]. Ascorbic peroxidase (APX) activity was measured following the commercial assay kit (Beijing Solarbio Technology Company, China) according to the supplier’s protocol.

### Protein extraction and 2-DE separation

Proteins from storage roots of QZ1 and SC9 genotypes during PPD programme were extracted with phenol extraction according to Chen *et al*. (2009) [32]. Two-dimensional electrophoresis (2-DE) was carried out according to the method of An *et al*. (2014) [33]. Immobilized linear pH gradient strips (pH 4–7, 13 cm, GE Healthcare, UK) were loaded with 312 μl rehydration buffer containing 300 μg sample proteins at room temperature in tray for 12–16 h. Isoelectric focusing (IEF) was carried out using GE Healthcare Isoelectric Focusing System (GE Healthcare, UK) under the following conditions: 300 V for 0.05 h in gradient mode, 300 V for 0.10 h in step and hold, 3500 V for 1.30 h in gradient mode, 3500 V for 4.20 h in step and hold, and 300 V for 20 min in gradient mode at 20°C. Sodium dodecyl sulfate-polyacrylamide gel electrophoresis (SDS-PAGE) was carried out with 12% acrylamide gels. The resultant 2-DE gels were stained with Colloidal Coomassie Blue G-250 for 2–3 days. The stained gels were scanned by Image Scanner III (GE healthcare) and analyzed with Delta2D (DECODON GmbH, Greifswald, Germany) software. Relative comparison of the intensity abundance between control and treatments (three replicate samples for each group) was performed using the Scheffe’s test (P≤0.05). The protein spots with at least 2-fold change were considered to be differentially expressed proteins (DEPs).

### Tryptic in-gel digestion and MALDI-TOF/TOF MS analysis

Tryptic in-gel digestion and protein identification were performed by the methods reported in An *et al*. (2016) [34]. Differential proteins were identified using MALDI-TOF-TOF-MS/MS at Analysis and Testing Center, Jiangsu University and Beijing Genome Institute (Shenzhen). The mass spectra were acquired on a Matrix-assisted Laser Desorption ionization tandem time of flight mass spectrometer (Ultraflex-TOF-TOF, German). The MS spectra were searched against the NCB (http://www.ncbi.nlm.nih.gov) and cassava databases (http://phytozome.jgi.doe.gov/pz/portal.html#!search) using the MASCOT version 2.2.03 (http://www.matrixscience.com). Peptide mass tolerance was set as 0.3 Da and MS/MS ion mass tolerance was set at 0.15 Da, one missed cleavage was allowed, carbamidomethylation of cysteine as a fixed modification, and oxidation of methionine as a variable modification. Routine protein identification required sequence-confirmed data for a minimum of two peptides with recognition as the top ranking match in the Mascot Standard scoring system [33]. The proteins ID in NCBI database was changed according to cassava database in Phytozome. The classification analysis of differential proteins was according to the gene ontology (http://phytozome.jgi.doe.gov/pz/portal.html#!search).

### Generation of protein-protein interaction (PPI) networks

DEPs of cassava SC9 and QZ1 associated with PPD were submitted to search tool for the retrieval of their corresponding interaction genes (STRING). All interactions in STRING were provided with a probabilistic combined score 0.4. PPI network was constructed at String online software (http://string-db.org/newstring_cgi/show_input_page.pl) and metabolically function at PMN (http://pmn.plantcyc.org/CASSAVA/NEW-IMAGE?type=GENE&object=G2Z-8222&redirect=T). The proteins in the PPI network considered as nodes and the degree of a node corresponded to the number of interaction with other proteins. The proteins with high degrees were serves as hub nodes.

### Detection of quantitative real-time PCR (qRT-PCR)

The expressions of PPD responsive genes were validated by qRT-PCR with RNA samples extracted with a RNAprep Pure Plant Plus Kit according to the supplier’s protocol (TIANGEN, Code: DP441). The RNA quality was determined by running an agarose gel with GelStain (TransGen, Biotech, Code: GS101-01) staining. The RNA concentration was determined with NanoVue™ Plus ultramicro spectrophotometer (GE Healthcare). Reverse transcription was performed according to the manufacturer’s protocol (TransGen, Biotech, Code: AT311-02). Each cDNA sample was diluted 10 times in sterile ddH_2_O, and 1μl of this dilution was used as a template for real-time RT-PCR. The primers were listed in S1 Table. The real-time RT-PCR reactions were performed in a 10 μl volume containing 5 μl of 2 × SYBR^®^ Premix Ex TaqTM II (Tli RNaseH Plus) (TaKaRa, Code: RR820A), 1 μl (100 ng/μl) cDNA, and 0.8 μl (10 μM of each primer) primers in a Thermo Scientific PikoREAL thermocycler. Quantification was performed by sample of target genes to beta-actin gene using the comparative Ct method. The ΔCt was calculated by subtracting the average Ct of each treatment stage from the average Ct of beta-actin. The ΔΔCt was calculated by subtracting the ΔCt of each treatment stage from the ΔCt of the 0 h stage. The formula 2^-ΔΔCt^ was used to calculate the relative fold change between the treatment stages [35]. All of the samples were measured in triplicate, and the experiments were performed on three biological replicates.

### Statistical analysis

All the data were represented by an average of at least three biological replicates, with error bars representing standard deviations. Data were analyzed using ANOVA, which was performed by using SPSS Statistics V20 software to Duncan’s multiple comparison tests. Significance was determined at the 0.05 level.

## Results

### Changes in dry matter, starch and β-carotene contents in cassava injured storage roots

**Fig 1:**
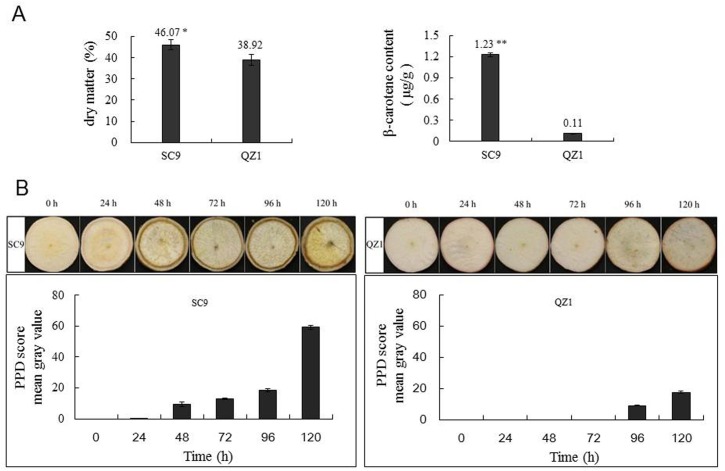
(A) The analysis of dry matter and β-carotene contents of cassava injured storage roots in both genotypes SC9 and QZ1. Values are the means ± SE from three biological replicates. * and ** indicate significant differences at P < 0.05 and 0.01, respectively, by SPSS Duncan’s multiple comparison tests. (B) PPD score analysis of injured storage root in SC9 and QZ1 genotypes after postharvest. Visual examination of SC9 and QZ1 storage root slices at the time-points from 0h to 120h. PPD scores at different time-points were obtained using ImageJ image processing software based on the mean gray values percentages of vascular discoloration. The mean gray value at 0h was set to 0.

As showed in Fig 1 A, the dry matter and beta-carotene of QZ1 genotype were 38.92% and 0.11 μg/g, respectively, They were significantly lower than those in SC9 genotype (46.07%, 1.23 μg/g), especially, the differences in β-carotene content reached a significant level (p<0.01) between SC9 and QZ1 genotypes. In order to visually evaluate the PPD phenomenon, the visions of injured cassava storage roots were observed in the present study. Fig 1B showed that the PPD symptom score in the injured storage root of QZ1 genotype was 0–17.5% during 120h, while in SC9 was 0–59.2%, suggesting that QZ1 could have significantly PPD tolerance higher than that of SC9.

### Analysis of H_2_O_2_ content and enzyme activities during PPD

**Fig 2:**
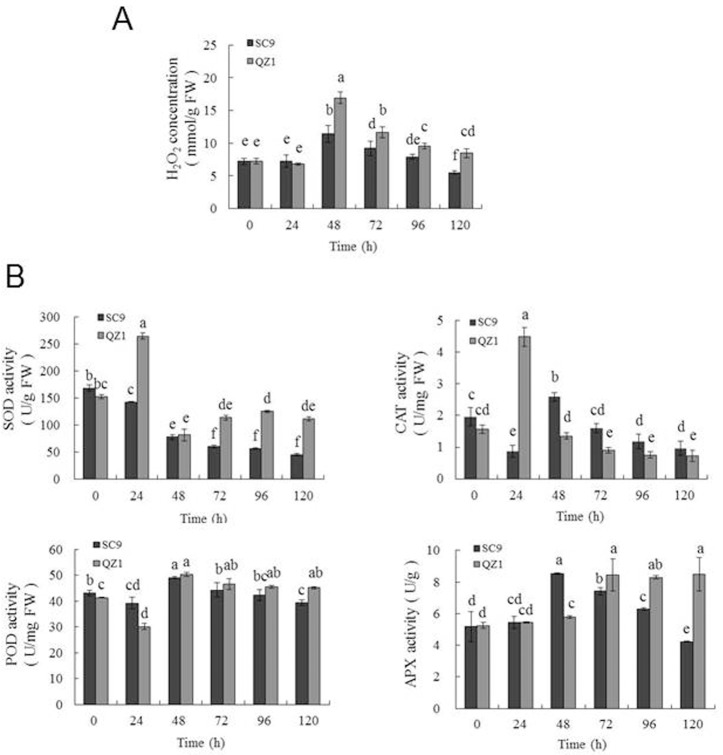
Changes in H_2_O_2_ contents and enzymatic activities at the time-points from 0h to 120h in two cassava genotypes. (A) H_2_O_2_ contents (mmol/g of fresh weight); (B) SOD activities (U/g of fresh weight), CAT activities (U/mg of fresh weight), POD activities (U/mg of fresh weight), and APX activities (U/g). Each bar represents the mean of three independent replicates with standard error. Different letters on the columns indicate the statistical difference at p< 0.05 by SPSS to Duncan’s multiple comparison tests.

Antioxidant enzymes play important roles in ROS scavenging and minimizing oxidative damage under stress conditions [36]. In plant systems, the major initial sources of ROS during normal metabolism are the production of superoxide (O_2_^•−^ and H_2_O_2_, which can modulate ROS in the PPD response [13, 37, 38]. In the present study, the concentrations of H_2_O_2_ (Fig 2A), which is considered as the major ROS in plants, were detected to be highest in the time-point of 48h in both genotypes (SC9, 11.4 mmol/g FW; QZ1, 16.92 mmol/g FW) under 120h-injured storage roots, and then gradually dropped to a low amount, while the H_2_O_2_ content in QZ1 was higher than that in SC9 at the time-points from 48h to 120h (Fig 2A). The activities of SOD, POD and APX (Fig 2B) in QZ1 genotype were higher than those in SC9 genotype, which was consistent with the changes of H_2_O_2_ concentration. These results indicated that QZ1 genotype produced H_2_O_2_ concentration more than that in SC9 genotype during injured storage roots, but the ability of ROS scavenging was also higher than that in SC9 genotype.

### Protein identification and statistical analysis

**Fig 3:**
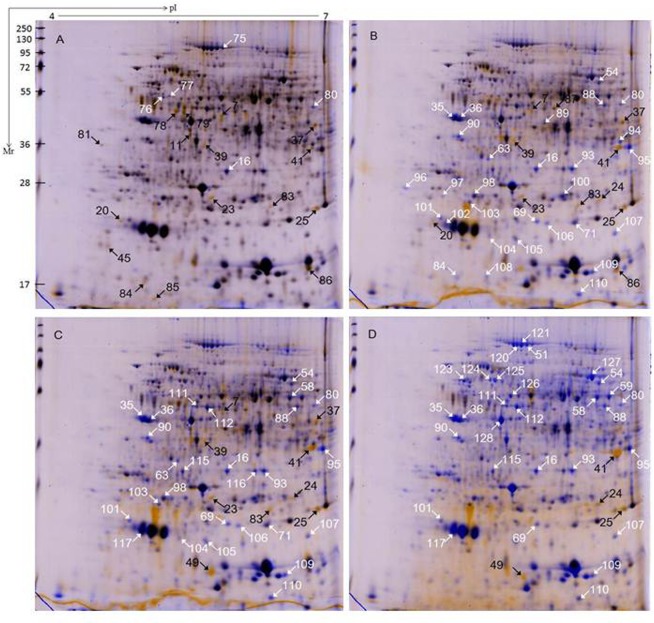
2-DE analysis of SC9 genotype at the time-points from 0h to 96h. Total of proteins (300 μg) were loaded on a 13 cm IPG strip with linear gradient (pH 4−7) and SDS-PAGE was performed on a 12% gel. Proteins were stained with CBB G-250. The wrapped 2-DE maps showed the pairwise comparison at the time-points of 24h/0 h (A), 48 h/0h (B), 72h/0h (C) and 96h/0h (D). The white and black arrows indicated proteins that showed detectable changes (>2.0-fold of the normalized volume) in abundance compared with those observed in the control of 0 h; white indicated a down-regulated match, and black indicated an up-regulated match.

**Fig 4:**
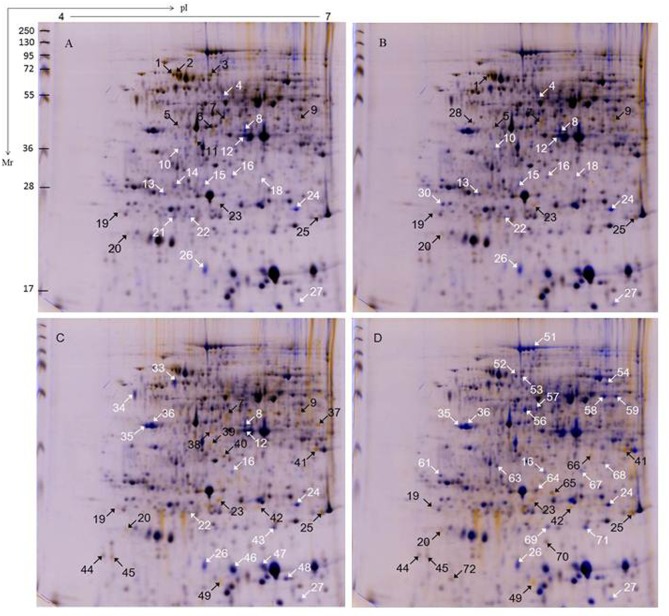
2-DE analysis of QZ1 genotype at the time-points from 0h to 96h. Total of proteins (300 μg) were loaded on a 13 cm IPG strip with linear gradient (pH 4−7) and SDS-PAGE was performed on a 12% gel. Proteins were stained with CBB G-250. The wrapped 2-DE maps showed the pairwise comparison at the time-points of 24h/0 h (A), 48 h/0h (B), 72h/0h (C) and 96h/0h (D). The white and black arrows indicated proteins that showed detectable changes (>2.0-fold of the normalized volume) in abundance compared with those observed in the control of 0 h; white indicated a down-regulated match, and black indicated an up-regulated match.

**Fig 5:**
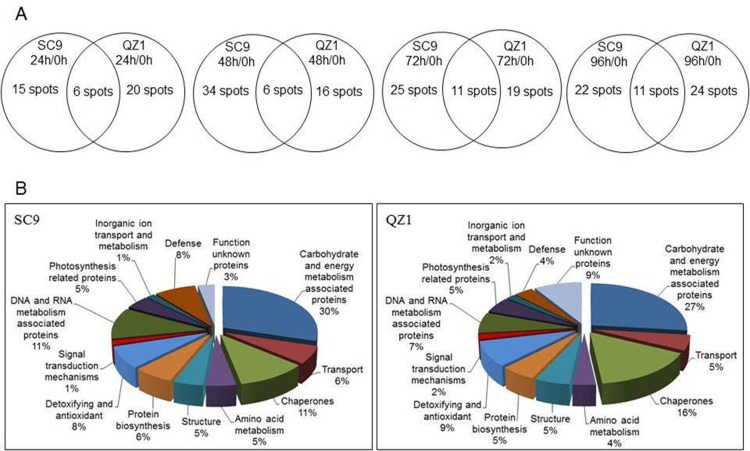
Venn diagrams and functional categories of differential proteins identified in cassava storage roots at the time-points from 0h to 96h. Unknown proteins included those whose functions had not been described. (A), venn diagrams of 24h/0h, 48h/0h, 72h/0h and 96h/0h in both genotypes (B), functional categories of 64 and 56 differential proteins identified in SC9 and QZ1 genotypes roots.

Proteins extracted from injured storage roots of two genotypes SC9 and QZ1 in response to PPD were separated by 2-DE. 2-DE images of proteins extracted from 0h time-point in the injured storage roots of SC9 and QZ1 genotypes were used as control, respectively. A total of 108 differential protein spots with greater than 2-fold altered intensity in the pairwise comparison of 24h/0h, 48h/0h, 72h/0h and 96h/0h were detected in both genotypes, in which 99 proteins were identified using MALDI-TOF-MS/MS. These identified proteins were involved in 13 biological functions including carbohydrate and energy metabolism (29), chaperones (15), DNA and RNA metabolism (9), detoxifying and antioxidant (8), defense (7), structure (6), transport (5), protein biosynthesis (5), amino acid metabolism (4), photosynthesis related proteins (4), signal transduction mechanisms (2), inorganic ion transport and metabolism (1) and function unknown proteins (6) (Table 1, S2 Table and S3 Table). Of these, 62 differential proteins were from SC9 genotype (Fig 3) and 56 differentially expressed proteins were identified from QZ1 genotype (Fig 4) against cassava database (https://phytozome.jgi.doe.gov/pz/portal.html#!info?alias=Org_Mesculenta) [25]. In both genotypes, there were 20 common proteins (CPs) (spots 7, 11, 16, 23–25, 35–37, 39, 41, 45, 49, 51, 54, 58, 59, 63, 69 and 71) listed in Table 1. Of these identified proteins, they were annotated via the survey of gene banks as shown in Table 1. There were 2 CPs (spots 16, 24) existed at the time-points of 24h, 48h, 72h and 96h; 2 CPs (spots 7, 23) at the former three time-points (24h, 48h and 72h); 1 CP (spot 25) at the latter three time-points (48h, 72h and 96h); 4 CPs (spots 35, 36, 41, 49) at the latter two time-points (72h and 96h) (Fig 5A). Carbohydrate and energy metabolism related proteins were the biggest amount in both genotypes (SC9, 30%; QZ1, 27%) (Fig 5B), followed by chaperones (SC9, 11%; QZ1, 16%), DNA and RNA metabolism associated proteins (SC9, 11%; QZ1, 7%), and defense (SC9, 8%; QZ1, 4%) (Fig 5B).

**Table 1:** Identification of differential proteins in cassava genotypes SC9 and QZ1 at the time-points from 0h to 96h. The spots showing differential expression (> 2.0-fold of the normalized volume) were counted after gel analysis and manual editing with Delta2D software.

Spot number^a^	Description	Protein ID^b^	Theoretical pI/Mw (kDa)	Score^c^/No. of unique peptides matched^d^	Fold changes in the pairwise comparison of 24/0h, 48/0h, 72/0h and 96/0h in QZ1/SC9	Differential proteins in the pairwise comparison in QZ1	Differential proteins in the pairwise comparison in SC9	Protein ID in the Database of *Arabidopsis thaliana*
***Carbohydrate and energy metabolism associated proteins (29)***								
3	V-type proton ATPase catalytic subunit	cassava4.1_003676m	5.01/27.32	265/5	3.589±0.012(+)	24h/0h		VHA-A
10	Phosphoglycerate mutase(2,3-diphosphoglycerate-dependent)	cassava4.1_004733m	6.05/65.11	90/3	∞(-)	24h/0h, 48h/0h		iPGAM2
11*	UDP-glycosyltransferase 78D1-related	cassava4.1_007209m	6.05/45.30	104/4	2.399±0.083(+)/2.088±0.074(+)	24h/0h,	24h/0h	UGT78D2
21	triosephosphate isomerase, chloroplastic	cassava4.1_012016m	7.26/39.89	85/2	2.625±0.036(-)	24h/0h		TIM
27	N-acetyltransferase 9	cassava4.1_034206m	7.38/16.53	88/2	4.003±0.148(-)	24h/0h, 48h/0h, 72h/0h,96h/0h		AT2G32030
28	ADP ribosylation factor-related	cassava4.1_020559m	4.39/8.75	136/5	3.120±0.102(+)	48h/0h		TTN5
33	ATP synthase	cassava4.1_006420m	5.05/51.24	78/2	2.684±0.120(-)	72h/0h		VAB2
36*	pyruvate dehydrogenase E1 component subunit beta, mitochondrial	cassava4.1_010116m	6.04/40.22	306/2	2.586±0.017(-)/2.682±0.015(-)	72h/0h, 96h/0h	48h/0h, 72h/0h, 96h/0h	MAB1
39*	pyruvate dehydrogenase E1 component subunit beta, mitochondrial	cassava4.1_010116m	6.08/34.46	55/2	4.413±0.203(+)/∞(+)	72h/0h	24h/0h, 48h/0h, 72h/0h	MAB1
40	Uroporphyrinogen decarboxylase/Uroporphyrinogen-III carboxy-lyase	cassava4.1_009378m	6.71/40.63	66/2	2.134±0.018(+)	72h/0h		HEME2
43	Triose-phosphate isomerase/Triosephosphate mutase	cassava4.1_014432m	5.87/27.23	88/3	2.020±0.016(-)	72h/0h		TPI
51*	Glycogen phosphorylase/Polyphosphorylase	cassava4.1_002466m	5.09/109.63	83/3	3.101±0.171(-)/12.799±0.453(-)	96h/0h	96h/0h	AT3G29320
54*	ATP synthase subunit beta-1, mitochondrial-related	cassava4.1_004726m	5.95/59.86	430/3	6.296±0.110(-)/3.756±0.124(-)	96h/0h	48h/0h, 72h/0h, 96h/0h	AT5G08680
56	glucose-1-phosphate adenylyltransferase small subunit, chloroplastic	cassava4.1_005518m	5.16/57.58	117/2	5.752±0.135(-)	96h/0h		ADG1
57	ATP synthase subunit beta-1, mitochondrial-related	cassava4.1_004726m	5.95/59.86	387/3	∞(-)	96h/0h		AT5G08680
75	Glycogen phosphorylase/Polyphosphorylase	cassava4.1_002466m	5.26/108.52	56/3	2.563±0.116(-)		24h/0h	AT3G29320
77	enolase	cassava4.1_007673m	6.05/49.74	174/5	8.288±0.314(-)		24h/0h	LOS2
78	Transaldolase/Glycerone transferase	cassava4.1_007758m	4.76/42.25	83/5	4.060±0.107(+)		24h/0h	TRA2
81	methionine sulfoxide reductase	cassava4.1_016963m	6.05/23.02	86/5	2.286±0.025(+)		24h/0h	PMSR1
83	Triose-phosphate isomerase/Triosephosphate mutase	cassava4.1_014432m	5.35/28.42	254/3	6.983±0.216(+)		24h/0h, 48h/0h,72h/0h	TPI
87	ATP synthase subunit beta-1, mitochondrial-related	cassava4.1_004726m	5.95/59.86	643/3	∞(+)		48h/0h	AT5G08680
88	cinnamyl alcohol dehydrogenase 6-related	cassava4.1_010153m	6.05/38.30	63/2	3.984±0.115(-)		48h/0h, 72h/0h, 96h/0h	CAD6
89	ATP synthase subunit beta-1, mitochondrial-related	cassava4.1_004726m	5.95/59.86	342/3	2.713±0.126(-)		48h/0h	AT5G08680
102	pheophorbide a oxygenase, chloroplastic	cassava4.1_005194m	6.68/53.93	53/5	3.349±0.164(-)		48h/0h	ACD1
111	ATP synthase subunit beta-1, mitochondrial-related	cassava4.1_004726m	6.00/59.93	103/3	∞(-)		72h/0h, 96h/0h	AT5G08680
115	Malate dehydrogenase (oxaloacetate-decarboxylating) (NADP(+))/Pyruvic-malic carboxylase	cassava4.1_004164m	7.92/26.71	97/2	2.421±0.117(-)		72h/0h, 96h/0h	NADP-ME2
120	Glycogen phosphorylase/Polyphosphorylase	cassava4.1_002466m	5.26/108.52	65/3	8.946±0.314(-)		96h/0h	AT3G29320
121	Glycogen phosphorylase/Polyphosphorylase	cassava4.1_002466m	5.26/108.52	70/3	13.243±0.463(-)		96h/0h	AT3G29320
126	ATP synthase subunit beta-1, mitochondrial-related	cassava4.1_004726m	6.00/ 59.12	88/3	4.503±0.135(-)		96h/0h	AT5G08680
***Transport (5)***								
25*	abscisic acid 8'-hydroxylase/ABA 8'-hydroxylase	cassava4.1_022491m	9.38/51.66	28/2	32.747±0.538(+)/5.369±0.146(+)	24h/0h, 48h/0h, 72h/0h, 96h/0h	24h/0h, 48h/0h, 72h/0h, 96h/0h	CYP707A4
46	Ethanolamine-phosphate cytidylyltransferase/Phosphorylethanolamine transferase	cassava4.1_008241m	6.68/53.93	67/3	2.049±0.113(-)	72h/0h		AT1G77060
71*	myosin	cassava4.1_000217m	4.79/200.17	44/3	2.624±0.096(-)/2.461±0.108(-)	96h/0h	48h/0h, 72h/0h	CIP1
94	Mitochondrial outer membrane translocase complex, subunit Tom7	cassava4.1_020644m	10.62/8.20	122/5	∞(-)		48h/0h	AT5G41685
116	dynactin sununit P25	cassava4.1_013839m	5.78/29.50	331/3	5.501±0.148(-)		72h/0h	GAMMACA1
***Chaperones (15)***								
1	heat chock protein 70KDa	cassava4.1_001607m	5.53/72.35	86/2	2.113±0.087(+)	24h/0h, 48h/0h		Hsp70b
2	heat shock 70 KDa protein 10, mitochondrial	cassava4.1_002964m	4.88/69.39	83/5	2.827±0.069(+)	24h/0h		HSC70-1
4	chaperonin	cassava4.1_004458m	5.50/60.52	107/5	∞(-)	24h/0h, 48h/0h		HSP60
45*	regulator of chromosome condensatio(RCC1) family with fyve zinc finger domain-related	cassava4.1_000603m	4.37/34.27	236/2	3.400±0.118(+)/4.112±0.136(+)	72h/0h, 96h/0h	24h/0h	BRX
47	17.6 KDa class I heat shock protein 1-related	cassava4.1_017974m	6.00/17.96	86/3	2.388±0.085(-)	72h/0h		AT1G53540
52	heat shock 70 Kda protein 10, mitochondrial	cassava4.1_002955m	5.35/73.40	117/2	2.738±0.097(-)	96h/0h		MTHSC70-2
53	heat shock 70 Kda protein 10, mitochondrial	cassava4.1_002964m	6.34/73.40	95/5	2.244±0.082(-)	96h/0h		MTHSC70-2
70	22.0 KDa heat chock protein	cassava4.1_033525m	6.05/21.51	142/3	2.471±0.079(+)	96h/0h		ATHSP22.0
72	annexin D1-related	cassava4.1_021183m	6.81/36.20	83/2	3.736±0.117(+)	96h/0h		ANNAT1
100	20 KDa chaperonin, chloroplastic	cassava4.1_014410m	8.87/27.76	341/5	2.120±0.068(-)		48h/0h	CPN20
104	25.3 KDa heat shock protein, chloroplastic	cassava4.1_015256m	7.26/25.96	89/3	7.025±0.264(-)		48h/0h,72h/0h	HSP21
105	22.0 KDa heat shock protein	cassava4.1_033525m	6.05/28.05	249/3	4.240±0.131(-)		48h/0h	ATHSP22.0
110	17.6 KDa class I heat shock protein 1-related	cassava4.1_018134m	5.13/1796	132/5	44.884±0.597(-)		48h/0h, 72h/0h, 96h/0h	AT1G07400
117	25.3 KDa heat shock protein, chlorplastic	cassava4.1_015256m	8.39/26.26	92/5	36.764±0.543(-)		72h/0h, 96h/0h	HSP21
124	chaperonin 60 subunit beta 1, chloroplastic-related	cassava4.1_003907m	5.42/11.40	215/5	∞(-)		96h/0h	CPN60B
***Amino acid metabolism (4)***								
13	aspartic proteinase A1-related	cassava4.1_005735m	4.73/56.41	88/5	2.874±0.122(-)	24h/0h, 48h/0h		APA1
23*	Phosphatidylserine decarboxylase/PS decarboxylase	cassava4.1_025763m	6.11/73.51	74/2	10.605±0.267(+)/∞(+)	24h/0h, 48h/0h, 72h/0h, 96h/0h	24h/0h, 48h/0h, 72h/0h	PSD2
85	ubiquitin-conjugating enzyme E2 1-related	cassava4.1_018317m	9.59/23.04	85/2	10.822±0.301(+)		24h/0h	UBC2
90	diaminopimelate epimerase,chloroplastic	cassava4.1_009997m	8.71/45.83	97/3	∞(-)		48h/0h, 72h/0h, 96h/0h	AT3G53580
***Structure(6)***								
5	actin	cassava4.1_033108m	5.52/39.83	81/2	2.633±0.085(+)	24h/0h, 48h/0h		ACT7
8	actin family protein	cassava4.1_009807m	5.16/41.89	37/4	3.621±0.106(-)	24h/0h, 48h/0h, 72h/0h		ACT7
12	ACT7; actin 7	cassava4.1_009934m	5.05/41.25	34.8/4	2.134±0.105(-)	24h/0h, 48h/0h, 72h/0h, 96h/0h		ACT7
76	tubulin beta-4 chain-related	cassava4.1_007713m	5.12/50.11	153/4	2.289±0.076(-)		24h/0h	TUB8
79	actin	cassava4.1_033108m	5.31/41.70	78/2	3.199±0.142(+)		24h/0h	ACT7
128	actin	cassava4.1_033108m	5.16/41.88	131/5	12.864±0.257(-)		96h/0h	ACT7
***Protein biosynthesis (5)***								
7*	elongation factor family protein	cassava4.1_003058m	6.12/46.93	135/4	8.593±0.215(+)/16.389±0.266(+)	24h/0h, 48h/0h, 72h/0h	24h/0h, 48h/0h, 72h/0h	RABE1b
15	CCAAT-binding transcription factor-related	cassava4.1_026597m	7.03/20.10	90/4	2.119±0.105(-)	24h/0h, 48h/0h		NF-YB5
49*	translation initiation factor 5A-related	cassava4.1_018059m	5.87/21.99	56/5	∞(+)/15.243±0.364(+)	72h/0h, 96h/0h	72h/0h, 96h/0h	ELF5A-1
98	asparagine synthetase	cassava4.1_014428m	5.30/22.97	73/4	2.431±0.086(-)		48h/0h, 72h/0h	AILP1
123	protein disulfide isomerase	cassava4.1_008355m	5.16/56.38	93/5	13.184±0.249(-)		96h/0h	PDIL1-1
***Detoxifying and antioxidant (8)***								
16*	L-ascorbate peroxidases, chloroplastic/mitochondrial-related	cassava4.1_009867m	9.59/42.17	67/5	4.850±0.147(-)/18.851±0.328(-)	24h/0h, 48h/0h, 72h/0h,96h/0h	24h/0h, 48h/0h, 72h/0h, 96h/0h	TAPX
22	proteasome subunit beta type	cassava4.1_011091m	7.06/31.53	116/4	∞(-)	24h/0h, 48h/0h, 72h/0h		AT3G26340
24*	L-ascorbate peroxidase 2, cytosolic	cassava4.1_013461m	6.96/26.70	143/3	9.216±0.251(-)/6.096±0.157(+)	24h/0h, 48h/0h, 72h/0h, 96h/0h	48h/0h, 72h/0h, 96h/0h	APX2
61	Dehydrin (Dehydrin)	cassava4.1_015875m	5.28/24.37	109/2	2.653±0.098(-)	96h/0h		ERD10
64	L-ascorbate peroxidase 2, cytosolic	cassava4.1_013461m	5.31/27.67	173/3	2.306±0.102(-)	96h/0h		APX1
103	ferritin	cassava4.1_013978m	5.48/26.06	75/4	∞(-)		48h/0h, 72h/0h	FER1
112	monodehydroascorbate reductase, cytoplasmic isoform 1-related	cassava4.1_007980m	6.53/54.02	92/5	5.837±0.176(-)		72h/0h, 96h/0h	MDAR1
127	NADP-dependent malic enzyme 2-related	cassava4.1_004170m	8.39/27.65	173/5	28.623±0.428(-)		96h/0h	NADP-ME2
***Signal transduction mechanisms (2)***								
30	14-3-3-like protein GF14 lambda	cassava4.1_014519m	4.82/29.44	169/2	2.138±0.084(-)	48h/0h		AT5G65430.3
86	ADP-ribosylation factor A1F	cassava4.1_016194m	9.68/16.69	74/3	6.225±0.208(+)		24h/0h, 48h/0h	ATARF
***DNA and RNA metabolism associated proteins (8)***								
26	Oxaloacetate decarboxylase/Oxaloacetate carboxy-lyase	cassava4.1_017832m	5.35/18.06	212/2	26.759±0.364(-)	24h/0h, 48h/0h, 72h/0h, 96h/0h		AT5G16450
37*	RNA-binding KH domain-containing protein	cassava4.1_002529m	7.53/112.26	89/5	11.450±0.266(+)/6.094±0.219(+)	72h/0h	24h/0h, 48h/0h, 72h/0h	HEN4
41*	Adenosylhomocysteine nucleosidase/S-adenosylhomocysteine/5'-methylthioadenosine nucleosidase	cassava4.1_021711m	6.52/36.25	71/5	∞(+)/14.019±0.241(+)	72h/0h, 96h/0h	24h/0h, 48h/0h, 72h/0h, 96h/0h	AT4G24350
48	Nucleolar RNA-binding protein NIFK	cassava4.1_018378m	7.85/16.85	135/2	2.090±0.096(-)	72h/0h		GRP7
80	60S ribosomal protein L4	cassava4.1_008812m	10.77/44.64	45/4	13.966±0.258(-)		24h/0h, 48h/0h, 72h/0h, 96h/0h	AT3G09630
84	leucine-rich repeat containing protein	cassava4.1_025603m	6.57/42.25	90/4	2.202±0.104(+)		24h/0h, 48h/0h	AT3G14470
96	zinc-finger of the FCS-type, C2-C2 (zf-FLZ)	cassava4.1_018370m	9.59/13.05	81/5	∞(-)		48h/0h	AT1G78020
107	ATDCL4,DCL4;dicer-like 4	cassava4.1_017435m	8.38/20.24	225/8	36.480±0.567(-)		48h/0h, 72h/0h, 96h/0h	DCL4
***Photosynthesis related proteins (3)***								
58*	ribulose bisphosphate carboxylase large chain-related	Manes.S113700	6.60/51.81	267/2	3.344±0.130(-)/10.700±0.241(-)	96h/0h	96h/0h	RBCL
59*	ribulose bisphosphate carboxylase large chain-related	Manes.S113700	6.60/51.81	436/3	3.304±0.149(-)	96h/0h		RBCL
63 *	Carboxyvinyl-carboxyphosphonate phosphorylmutase/CPEP phosphonomutase	cassava4.1_011697m	8.25/50.32	120/5	2.014±0.106(-)/2.023±0.111(-)	96h/0h	48h/0h	AT2G43180
***Inorganic ion transport and metabolism (1)***								
69*	calcium-binding protein CML15-related (CaM)	cassava4.1_034063m	4.22/17.53	78/3	2.732±0.077(-)/∞(-)	96h/0h	48h/0h, 72h/0h, 96h/0h	AT3G25600
***Defense (7)***								
18	thiamine thiazole synthase	cassava4.1_010620m	5.30/39.74	78/5	∞(-)	24h/0h		THI1
67	zinc finger protein ZAT2-related	cassava4.1_028217m	6.10/45.39	93/3	∞(-)	96h/0h		ZAT11
93	thiamine thiazole synthase	cassava4.1_010620m	5.30/34.37	94/5	23.471±0.420(-)		48h/0h, 72h/0h, 96h/0h	THI1
95	annexin D1-related	cassava4.1_021183m	6.81/36.20	59/2	14.957±0.364(-)		48h/0h, 72h/0h, 96h/0h	ANNAT1
97	ATFER2,FER2;ferritin 2	cassava4.1_014185m	5.1/29.47	134/5	∞(-)		48h/0h	FER2
101	Cysteine-rich TM module stress tolerance (CYSTM)	cassava4.1_020864m	9.27/27.56	146/5	4.182±0.146(-)		48h/0h, 72h/0h, 96h/0h	AT5G04080
109	RING/U-box superfamily protein	cassava4.1_017114m	8.17/20.92	270/12	∞(-)		48h/0h, 72h/0h, 96h/0h	AT3G10910
***Function unknown proteins (6)***								
34	RHO GDP-dissociation inhibitor	cassava4.1_015415m	5.16/27.29	82/2	2.042±0.076(-)	72h/0h		SCN1
35*	Plant protein of unknown function (DUF247)	cassava4.1_007729m	7.13/55.95	125/2	2.839±0.104(-)/8.282±0.259(-)	72h/0h, 96h/0h	48h/0h, 72h/0h, 96h/0h	AT3G50120
44	unnamed protein	cassava4.1_020024m	10.33/10.83	63/2	2.321±0.116(+)	72h/0h, 96h/0h		
66	Uncharacterized conserved protein	cassava4.1_020840m	9.86/7.84	89/2	44.827±0.572(+)	96h/0h		
68	Arabidopsis protein of unknown function (DUF241)	cassava4.1_028170m	8.27/33.01	147/3	2.009±0.083(-)	96h/0h		AT2G17080
106	Domain of unknown function (DUF4283) (DUF4283)//Zinc knuckle (zf-CCHC_4)	cassava4.1_025719m	7.26/13.05	146/3	∞(-)		48h/0h, 72h/0h	AT3G31430

a, The numbers corresponded to the 2-DE gel in Figs 3 and 4.

b, NCBI accession number.

c, Probability-based MOWSE (molecular weight search) scores.

d, The number of unique peptides identified by MALDI-TOF-MS/MS, and individual ions scores are all identity or extensive homology (p<0.05).

* indicates CP spots between SC9 and QZ1 genotypes. (+) means up-regulated compare with 0h, while (-) means down-regulated compare with 0h.

### PPI networks

**Fig 6:**
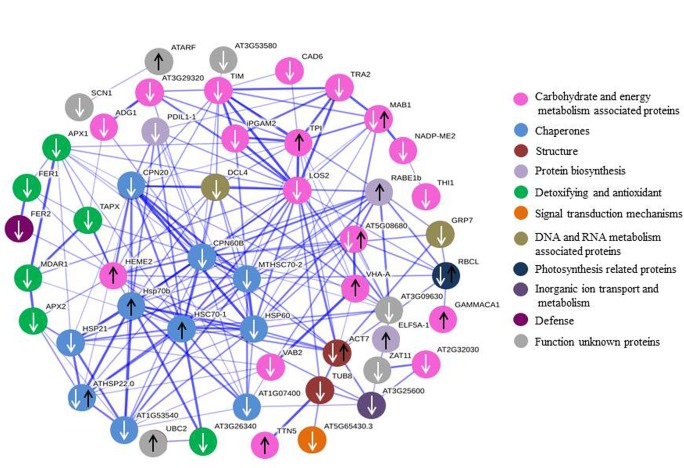
PPI network of differential proteins by string online software according to the database of *A*. *thaliana* PPI. White arrows indicate down-regulated proteins, and black arrows indicated up-regulated proteins. The different colour circle indicates the proteins with different biological functions. The network nodes are proteins, whereas the edges represent the predicted or known functional association.

The total proteins identified in each genotype were used to construct a PPI network by employing the STRING interface using the model plant *Arabidopsis thaliana* database [39]. The PPI network was constructed with 76 nodes and 196 edges (Fig 6), in which the proteins such as CPN60B (spot 124, chaperonin 60 subunit beta 1), LOS2 (spot 77, enolase), HSC70-1 (spot 2, heat shock 70 KDa protein 10) and CPN20B (spot 100, 20 KDa chaperonin) were viewed as hub proteins with a degree of 24, 21, 19 and 18 edges, respectively. In the PPI network, the proteins associated with carbohydrate and energy metabolism constituted the major nodes, followed by chaperones, and detoxifying and antioxidant.

### qRT-PCR analysis of gene expression

**Fig 7:**
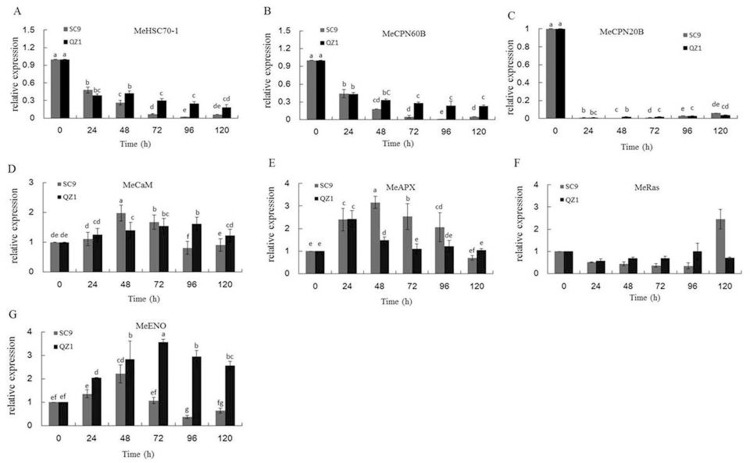
**The expressed levels of genes including *MeHSC70-1* (A), *MeCPN60B* (B), *MeCPN20B* (C), *MeCaM* (D), *MeAPX* (E), *MeRas* (F) and *MeENO* (G) in cassava injured storage roots using qRT-PCR.** Each bar represents the mean of three independent replicates with standard error. Different letters on the columns indicate the statistical difference at p< 0.05 by SPSS to Duncan’s multiple comparison tests.

Fig 7 showed the transcription levels of *MeHSC70-1*, *MeCPN60B*, *MeCPN20B*, *MeCaM*, *MeAPX*, *MeRas* and *MeENO* of injured storage roots in response to PPD using qRT-PCR. *MeHSC70-1*, *MeCPN60B*, *MeCPN20B* expression levels was down-regulated in SC9 and QZ1 genotypes (Fig 7A–7C). *MeCaM* expression levels in SC9 were higher than that of QZ1 at the time-points of 48h and 72h, and then decreased more than that in QZ1 (Fig 7D). The expression of *MeAPX* gene was higher than that of QZ1 after the time-point of 48h (Fig 7E). However, from the time-points of 24h to 96h, the expression of *MeRas* gene in QZ1 was higher than that in SC9 (Fig 7F). The expression level of gene *MeENO* in QZ1 genotype was higher than in SC9 at all time-points, in addition, the highest expressed level was observed at the time-points of 48h and 72h, respectively (Fig 7G).

## Discussion

PPD causes cassava storage root to be spoilage, and reduce the shelf-life in the cassava value chain. This phenomenon is a truly global challenge. Many breeders tried to develop solutions that increase the time it takes for cassava to become inedible or unfit processing following post-harvest, such as releasing PPD-tolerant cassava varieties to benefit smallholders and cassava-production manufactures. It was reported that PPD was negatively correlated with dry matter content in the roots [21, 22]. In addition, Chavez *et al*. (2000) reported 0.5μg/g β-carotene concentration was a threshold for cassava PPD tolerant varieties. When β-carotene concentration in cassava storage roots was higher than 0.5μg/g, the root PPD was always lower than 30% [40]. However, Morante et al (2010) mentioned that cassava MCol 2436 had the lowest levels of carotenoid (9.1 μg/g total carotenoid content) and considerable PPD damage. Perhaps there is a threshold effect and a minimum concentration of carotenoids (>9.1 μg/g) is required for their antioxidant properties to be effective [22]. It was reported that over 90% of the total carotenoids present in sweet yellow cassava were β-carotene [41]. In the present study, β-carotene contents were showed significant differences in SC9 (1.23μg/g) and QZ1 (0.11μg/g), but those data were much lower than 9.1 μg/g. QZ1 had higher PPD tolerance than that of SC9, suggesting β-carotene antioxidant property in SC9 was not effective. The result was consistent with the report from Morante et al (2010).

As previous reports indicated that PPD was associated with ROS production [13, 18, 19, 42]. PPD onset was mostly regulated by the balance between ROS and changes in the activities of antioxidant enzymes [38]. H_2_O_2_ is moderately reactive. It has a relatively long half-life and high permeability across membranes [16]. In the present study, H_2_O_2_ content increased apparently to the highest value in the injured storage roots of SC9 and QZ1 at the time-point of 48h (Fig 2A). SOD and CAT activities increased to the highest values in PPD-tolerant genotype QZ1 at the time-point of 24h; however, CAT and APX activities in PPD-susceptive genotype SC9 reached the higher values at the time-point of 48h (Fig 2B and 2C). SOD has been reported to work in collaboration with CAT which acted in tandem to remove H_2_O_2_ [43, 44]. It seems to show that the high activities of SOD and CAT antioxidant in QZ1 may be used to remove the increased H_2_O_2._ It means SOD in combination with CAT activities would be the first line of defense against PPD for the PPD-tolerant cassava variety, and could be used as a signaling to detect PPD phenomenon ahead of H_2_O_2_. The first line of defense against PPD in SC9 was weak and resulted in the production of PPD phenomenon at the time-point of 24h (Fig 1). POD was likely to participate with PPD onset because its activity was increased for the injured storage roots in response to PPD, whilst high tolerant cultivars exhibited lower level of POD activity during the post-harvest period [13]. This result was in accord with the data showed in the time-point of 24h of QZ1 in the present study. Xu *et al*. (2014) reported APX was used as simultaneously activated antioxidant to participate the defense mechanisms via cyclic ROS scavenging [45]. APX increased in the PPD-susceptible cultivars SAN and IAC to storage for 3d to produce PPD phenomenon, however, in the PPD-tolerant BRA cultivar for 5d to find PPD [46], suggesting APX may participate in the construction of the second line of defense in order to maintain the low levels of ROS produced from PPD. The second line of defense against PPD is the presence of endogenous antioxidant chemicals, such as other antioxidant enzymes [46–48].

Functional classification of the identified proteins from 2-DE images showed that the differential proteins in response to PPD were related to chaperones, DNA and RNA metabolism and defense in both genotypes. The identified proteins were also involved in ROS detoxification including APX1 (spots, 16, 24, 64), monodehydroascorbate reductase, cytoplasmic isoform 1-related (spot 112) and NADP-dependent malic enzyme 2-related (spot 127). APX was an important enzyme for detoxification of H_2_O_2_ in plants [49, 50]. Its expression was in response to diverse abiotic stress conditions. Overexpressing APX in chloroplasts in plants produced tolerant ability to salinity stress and drought conditions [51, 52]. However, in the present study, qRT-PCR data showed that APX expressions in SC9 were higher than that in QZ1 between the time-points of 48h and 96h (Fig 7D), which were consistent with the 2-DE data (Table 1). It may indicate that PPD-susceptible/tolerant (SC9/QZ1) genotypes all booted up the second line of defense against PPD at the time-point of 24h. APX expression in SC9 increased to a highest value at the time-point of 48h, but QZ1 decreased at the same time-point. These data showed that the first line of defense, consisting of SOD in combination with CAT activities, may play an important role against PPD in the PPD-tolerant genotype QZ1. This defense system could support the PPD tolerance in QZ1. The second line of defense, consisting of APX, and CaM, may work together against PPD in the PPD-susceptible SC9. This result was in accord with Owiti *et al*. (2011) reported [26].

In the previous reports, Ca^2+^- CaM may be linked with regulating PPD onset [53]. Followed the storage-root wounding, calcium changes preceded a burst in ROS [54, 55]. The Ca^2+^- CaM complex bound and activated a collection of target proteins leading to a physiological response [26]. Owiti *et al*. (2011) showed the expression of CaM played an important role in signal transduction under heat stress, and the expressions of several heat shock proteins (HSPs) were correlated with accumulation of CaM transcripts and proteins in plants [26]. In the present study, *CaM* expression in QZ1 was up-regulated at the time-points from 24h to 120h, but it was down-regulated in SC9 at the time-points of 96h and 120h. The homology of protein CaM in the present study compared with reported by Owiti reached 71.19% using DANMAN software. HSPs were down-regulated in SC9 genotype (spots, 100, 104, 105, 110, 117). In QZ1 genotype there were two up-regulated HSPs (spots, 1 and 2), four down-regulated HSPs (spots, 47, 52, 53, 70). Of these HSPs in PPI network, heat shock 70 KDa protein (spot 2) was recognized as hub protein (Fig 6). It was reported that the HSPs were involved in response to environmental stress such as heat, cold, drought and salinity stress [56, 57]. In plants, the HSPs have been speculated to act as an antioxidant under oxidative stress [58] or as an important tool for genetic manipulation of protein content in cassava storage root contributing to a natural sink for protein and carotenoid accumulation in intense yellow roots [59]. Further elucidation of the roles of HSPs in binding specifically with PPD would provide our understanding of the molecular machinery controlling PPD onset.

Ras GTPase binding protein is the essential negative regulator of the Ras signaling pathway [60] and induced by salt stress in smooth cordgrass [61]. ADP-ribosylation factor A1F (spot 86), a member of the ARF family of GTP-binding proteins of the Ras superfamily, involved in signal transduction. It is known that transcript accumulation is not always correlated with protein A1F synthesis. The differences between transcript and protein levels may be due to the mechanisms of control gene expression responsible by modulation of genes coding for proteins involving in PPD.

Enolase (ENO, spot 77) was found to have significantly increased oxidation. It is a metalloenzyme involved in the catalysis of 2-phosphoglycerate to phosphoenolpyruvate in the penultimate step of glycolysis [62]. Manaa *et al*. (2011) [63] reported that the enolase abundance was increased in both wild-type and stress tolerant the roots of tomato under salt stress. Enolase was increased in response to heat stress in rice [64] and flooding stress in soybean roots [65, 66]. These results indicated that enolase was an abiotic stress-responsive protein. In the present study, enolase, used as a hub protein, was highly decreased in abundance in the roots of the PPD-susceptible SC9 genotype during storage stages under injured stress. In addition, the gene *MeENO* transcriptional level in SC9 genotype was lower than in QZ1 (Fig 7G). These results suggest that balance of glycolysis metabolism might be involved in injured tolerance in cassava during storage stages.

## Conclusions

In the present study, QZ1 was resistant to PPD in storage roots compared to SC9, mainly due to the following two defense lines. SOD in combination with CAT activities would be the first line of defense against PPD for the PPD-tolerant cassava variety, The second line of defense, consisting of APX and CaM, may work together against PPD in the PPD-susceptible SC9. The 108 differential protein spots on the 2-DE gel image were detected in the injured storage roots stored at room temperature for 120h. Of these, 99 differential proteins were identified by MALDI-TOF-MS/MS. These identified proteins were involved in 13 biological functions. All differential proteins were used to generate the PPI network and 4 hub proteins including CPN60B, LOS2, HSC70-1 and CPN20B were speculated to be the candidate key proteins associated with PPD, which will provide insights into the improvement of cassava PPD-tolerant varieties and further analyze the PPD-tolerant mechanisms of cassava storage roots.

## Supporting information

S1 Table Primers used for qRT-PCR analysis. (DOCX)

S2 Table Differentially expressed proteins identified using MALDI-TOF/TOF- MS/MS in storage roots of SC9 genotype. (XLSX)

S3 Table Differentially expressed proteins identified using MALDI-TOF/TOF- MS/MS in storage roots of QZ1 genotype. (XLSX)

S1 Fig Three biological replicates of 2-D gels of SC9 at the time-points of 0h. (TIF)

S2 Fig Three biological replicates of 2-D gels of SC9 at the time-points of 24h. (TIF)

S3 Fig Three biological replicates of 2-D gels of SC9 at the time-points of 48h. (TIF)

S4 Fig Three biological replicates of 2-D gels of SC9 at the time-points of 72h. (TIF)

S5 Fig Three biological replicates of 2-D gels of SC9 at the time-points of 96h. (TIF)

S6 Fig Three biological replicates of 2-D gels of QZ1 at the time-points of 0h. (TIF)

S7 Fig Three biological replicates of 2-D gels of QZ1 at the time-points of 24h. (TIF)

S8 Fig Three biological replicates of 2-D gels of QZ1 at the time-points of 48h. (TIF)

S9 Fig Three biological replicates of 2-D gels of QZ1 at the time-points of 72h. (TIF)

S10 Fig Three biological replicates of 2-D gels of QZ1 at the time-points of 96h. (TIF)
