# Supplementary material for: Proteomic analysis of injured storage roots in cassava (Manihot esculenta Crantz) under postharvest physiological deterioration
Source: PLoS One. 2017 Mar 24;12(3):e0174238. doi: 10.1371/journal.pone.0174238 (PMC5365129; doi:10.1371/journal.pone.0174238)
Supplement: S1 Table — (DOCX) [file pone.0174238.s001.docx]

| S1 Table. Primers used for qRT-PCR analysis. | | | |  |
| --- | --- | --- | --- | --- |
|  | Gene name | Forward primer (5'-3') | Reverse primer (5'-3') | |
|  | *actin* | TGATGAGTCTGGTCCATCCA | CCTCCTACGACCCAATCTCA | |
| MeCaM | *calmodulin* | GGCAGAGCTCCAGGACATGA | GGCAGCAGCAGAAATAAAACCA | |
| MeAPX | *ascorbate peroxidase* | AAGGATATTGTTGTCCTTTCTGGTG | CAAAGATGAGAGGATTAGGAGTCCA | |
| MeRas | *Ran GTPase binding protein* | AAAGCGAGGTGGCATCCAAGTTAGA | ACTCGGGTGTTGGTTTTCTTTTCCT | |
| MeENO | *enolase* | AAAGAGGCTATGAAGATGGGTG | CAAAACCACCTTCATCACCAAC | |
| MeHSC70-1 | *heat shock 70 KDa protein 10* | GCACTGCTGGAGTTCTTAGTGAGT | CTTAGCCTTTTCTGCTGCTTCTCT | |
| MeCPN20 | *20 KDachaperonin* | ACAGTAGGAGAAGTACGAGCACCA | CTTCACGTCCTCCTTTGAGAGTC | |
| MeCPN60B | *chaperonin 60 subunit beta 1* | CTCAAGGTCTTATTGCTGAAGGTG | TCATTCACTAGACCCTTTGTGGTC | |
