# Supplementary figures and images for: Proteomic analysis of injured storage roots in cassava (Manihot esculenta Crantz) under postharvest physiological deterioration
Source: PLoS One. 2017 Mar 24;12(3):e0174238. doi: 10.1371/journal.pone.0174238 (PMC5365129; doi:10.1371/journal.pone.0174238)

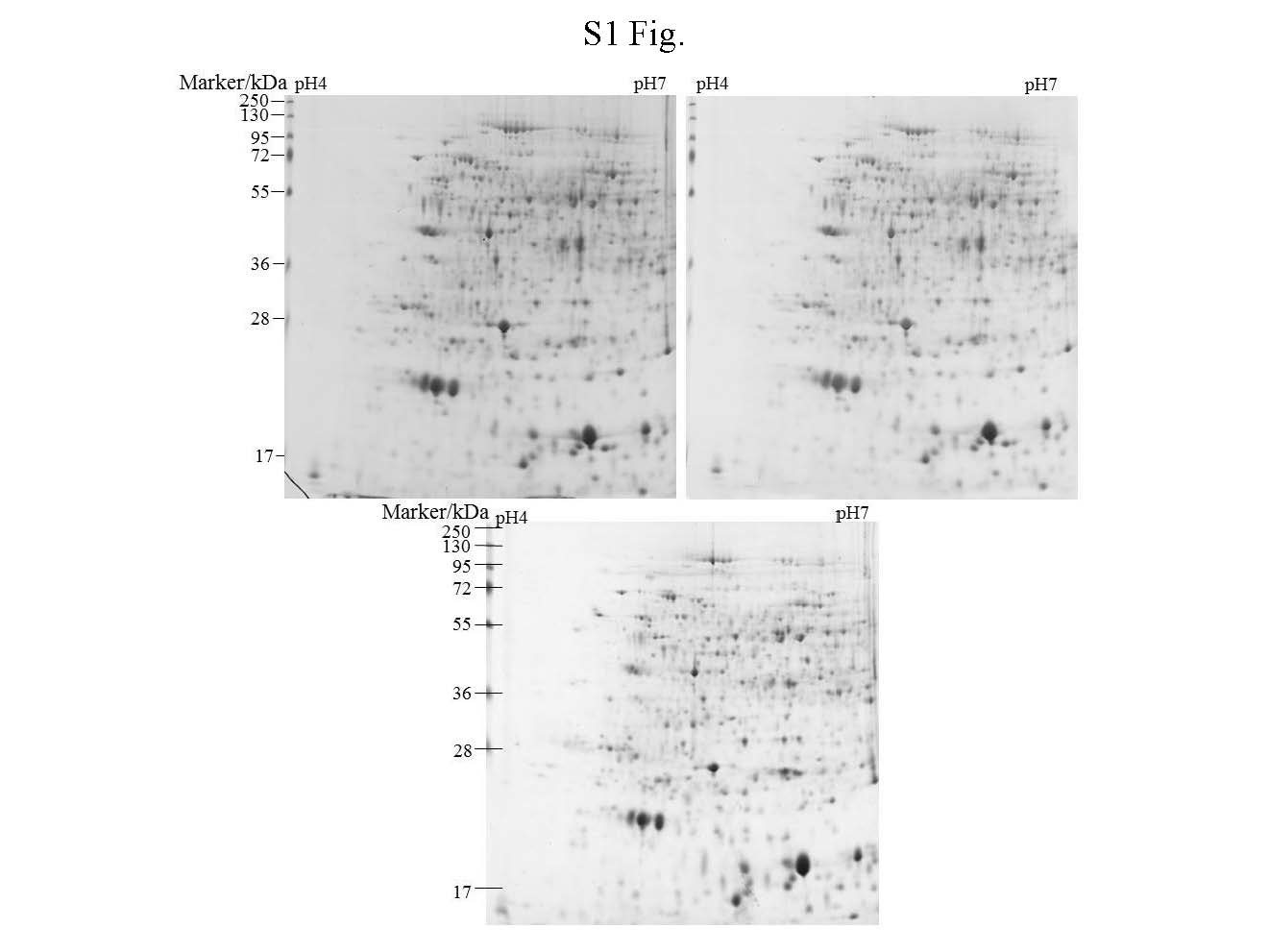

Supplement: S1 Fig — (TIF) [file pone.0174238.s004.tif]

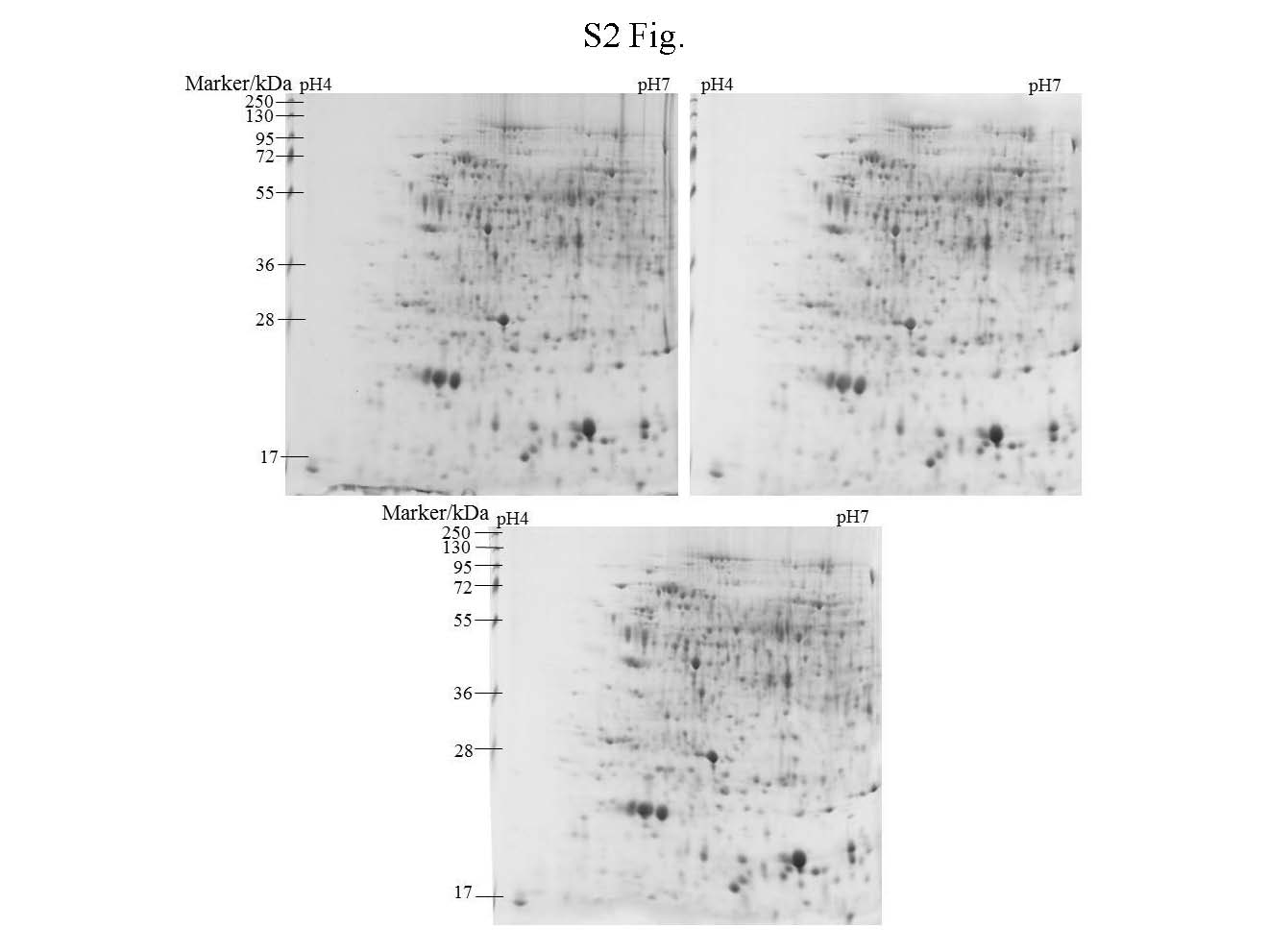

Supplement: S2 Fig — (TIF) [file pone.0174238.s005.tif]

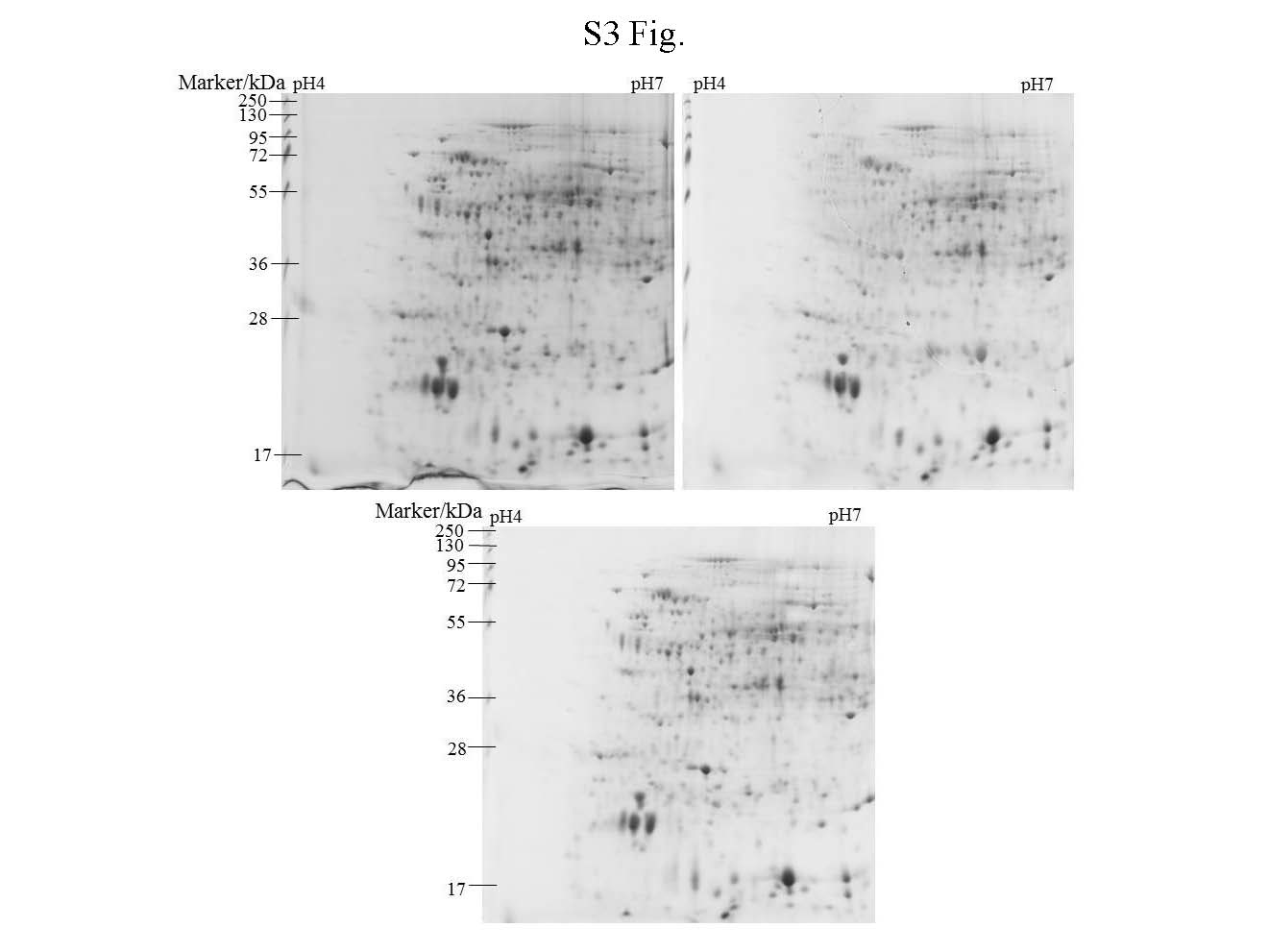

Supplement: S3 Fig — (TIF) [file pone.0174238.s006.tif]

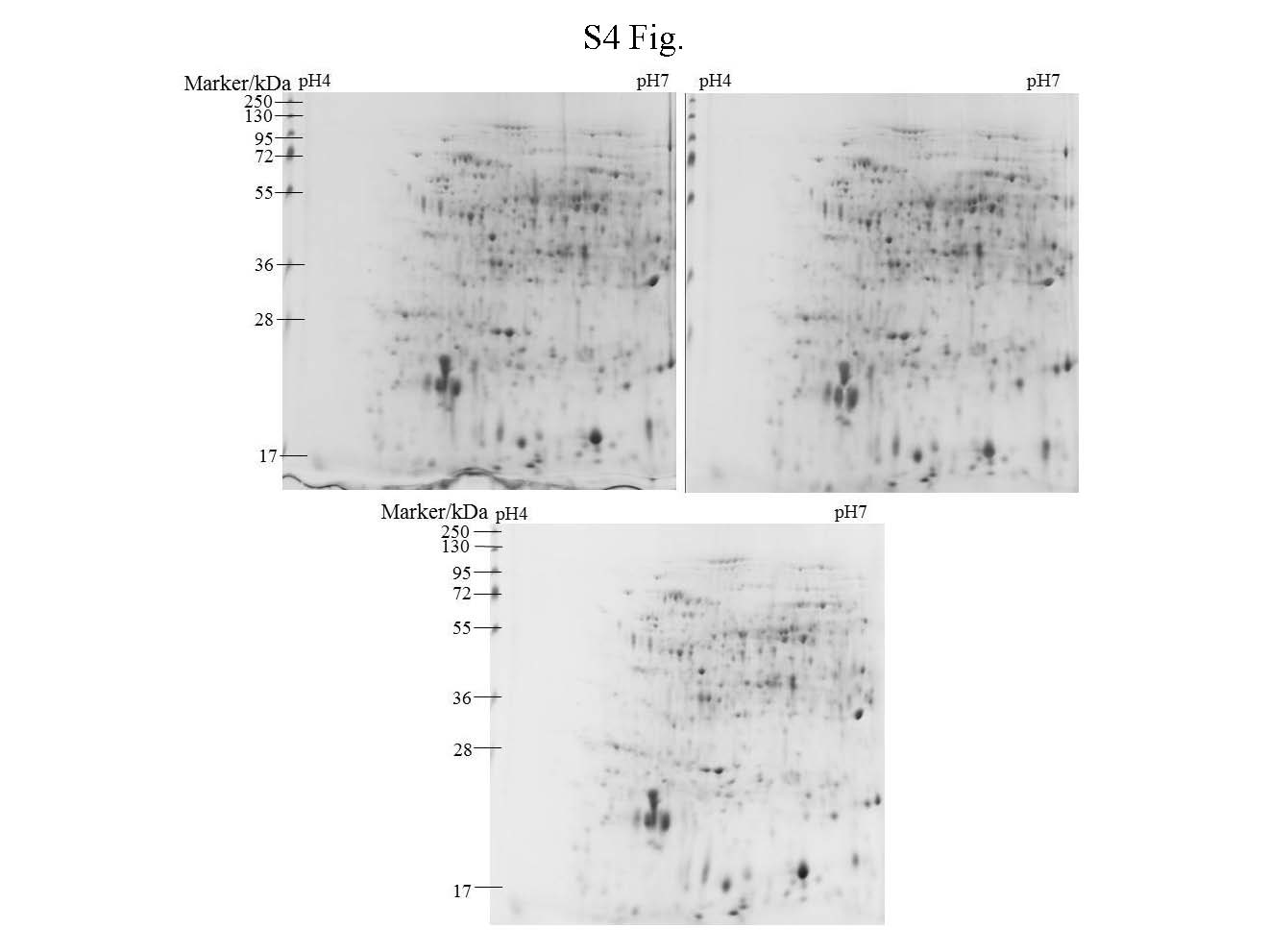

Supplement: S4 Fig — (TIF) [file pone.0174238.s007.tif]

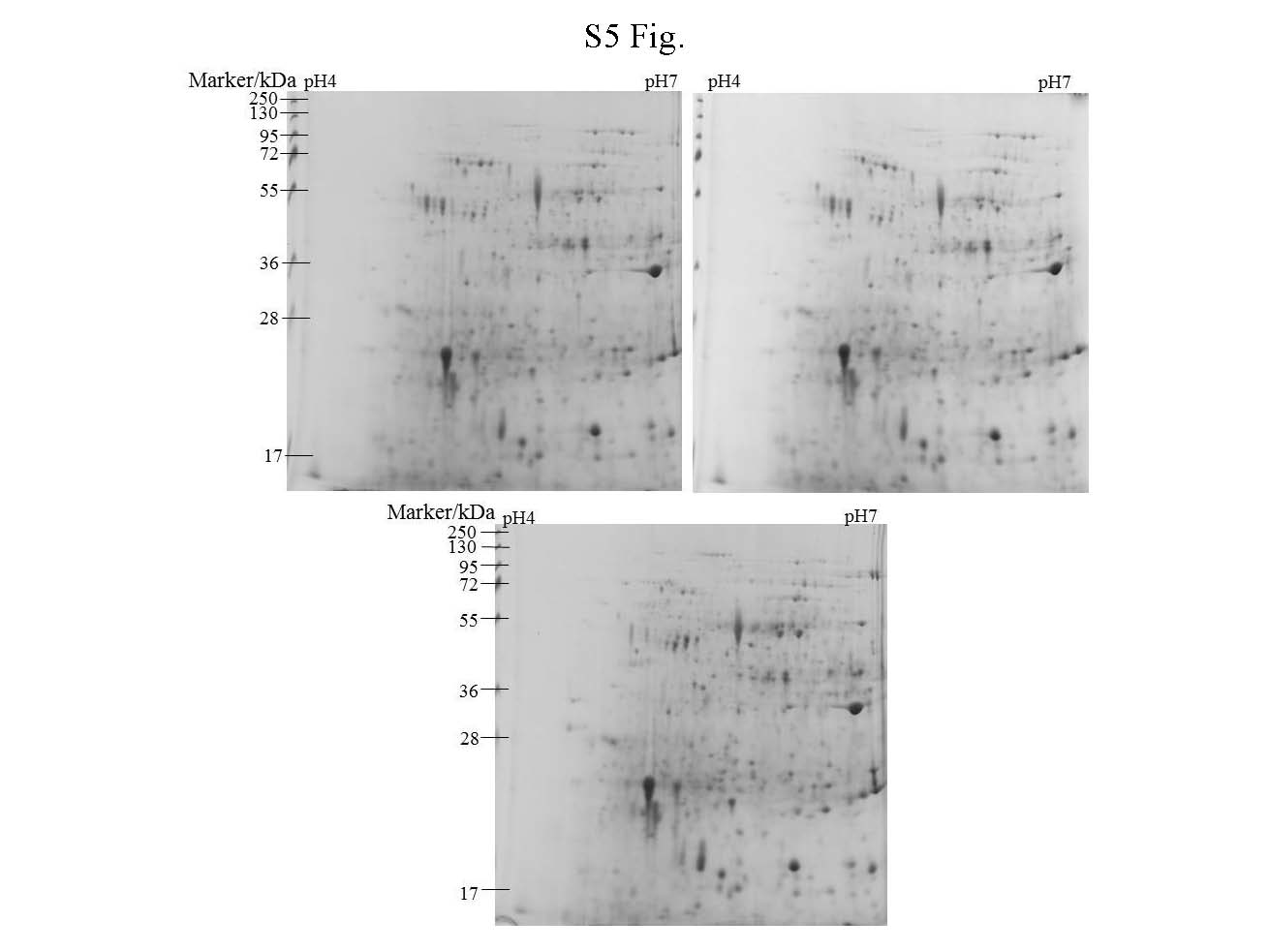

Supplement: S5 Fig — (TIF) [file pone.0174238.s008.tif]

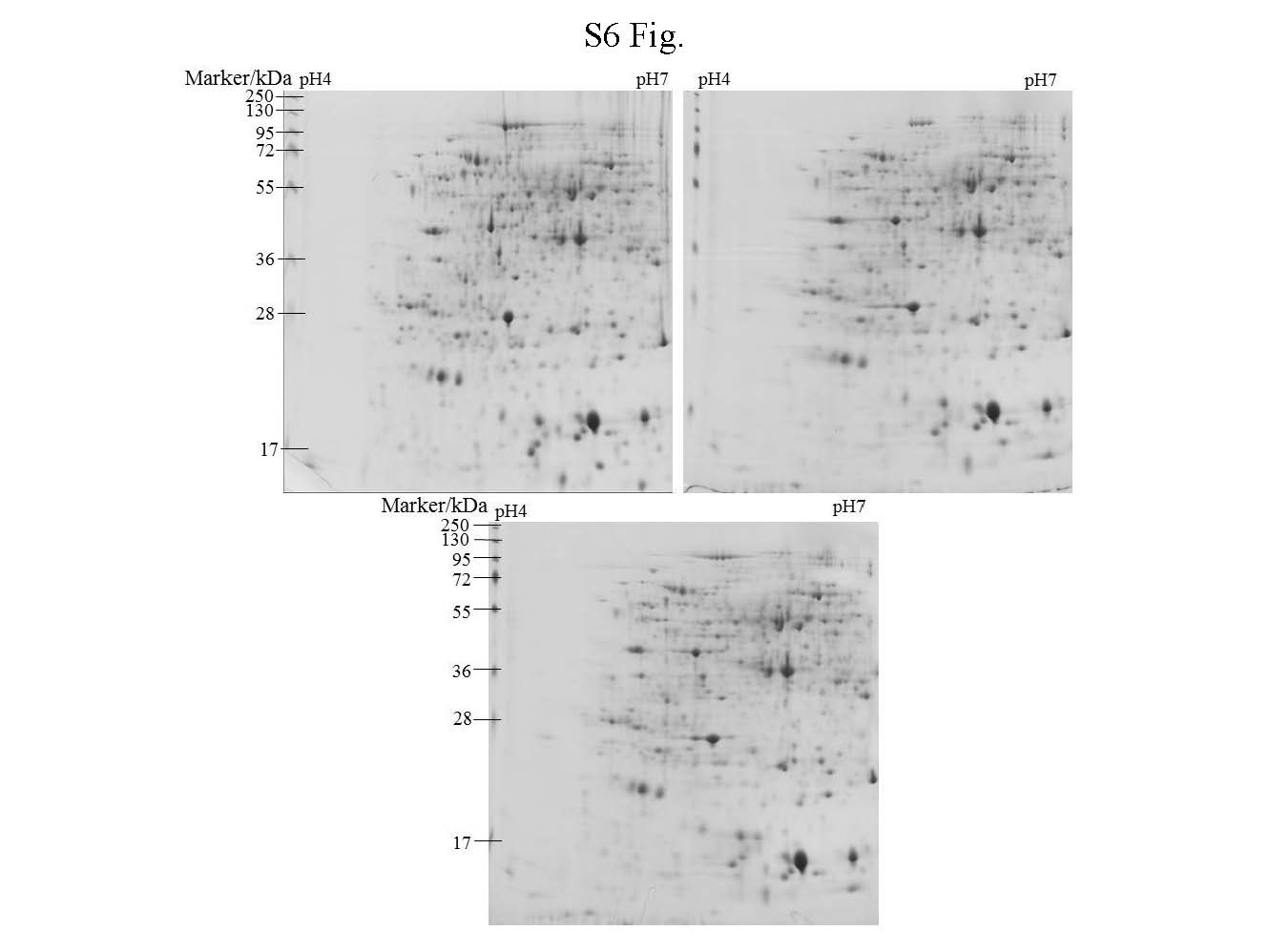

Supplement: S6 Fig — (TIF) [file pone.0174238.s009.tif]

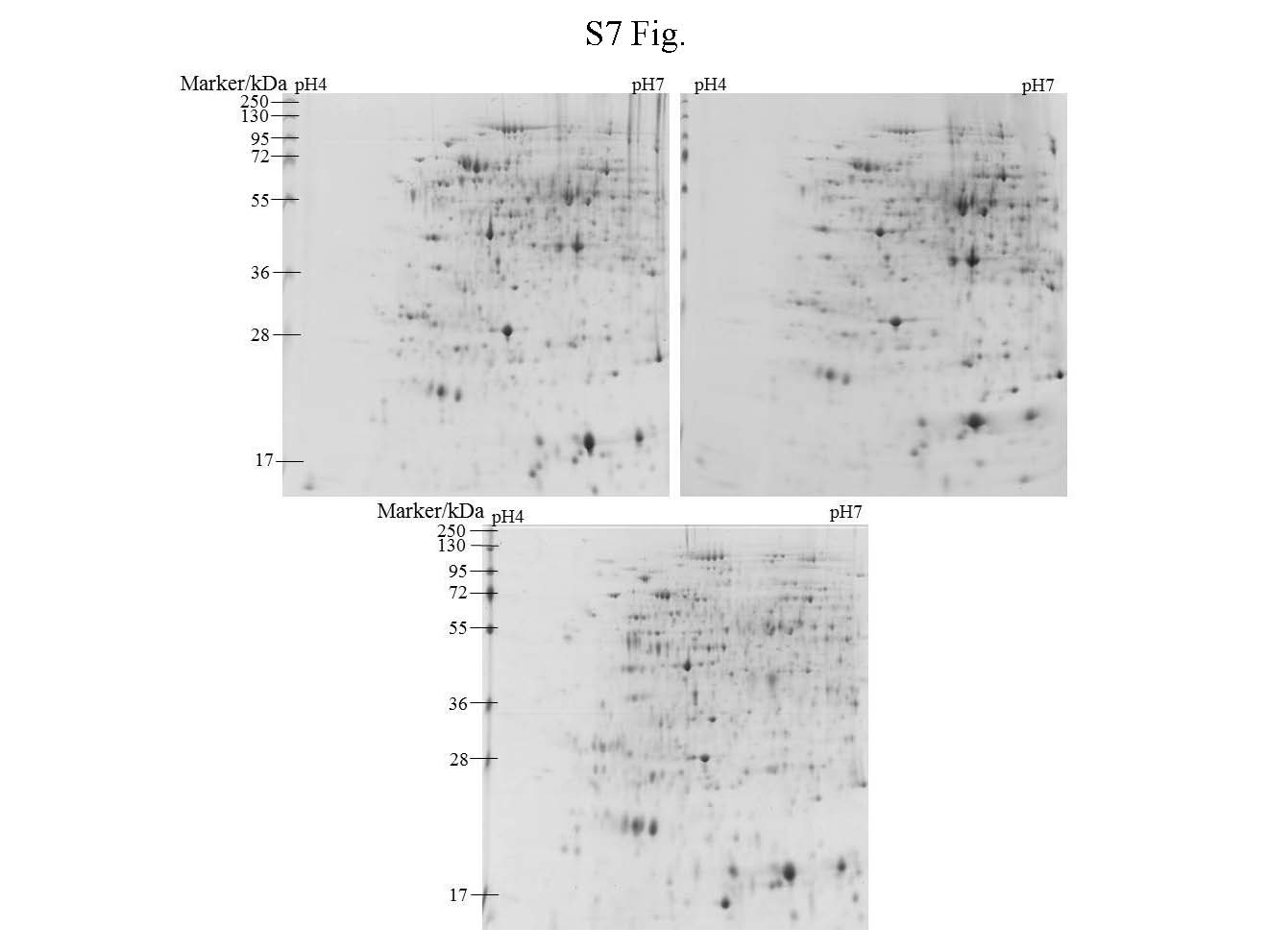

Supplement: S7 Fig — (TIF) [file pone.0174238.s010.tif]

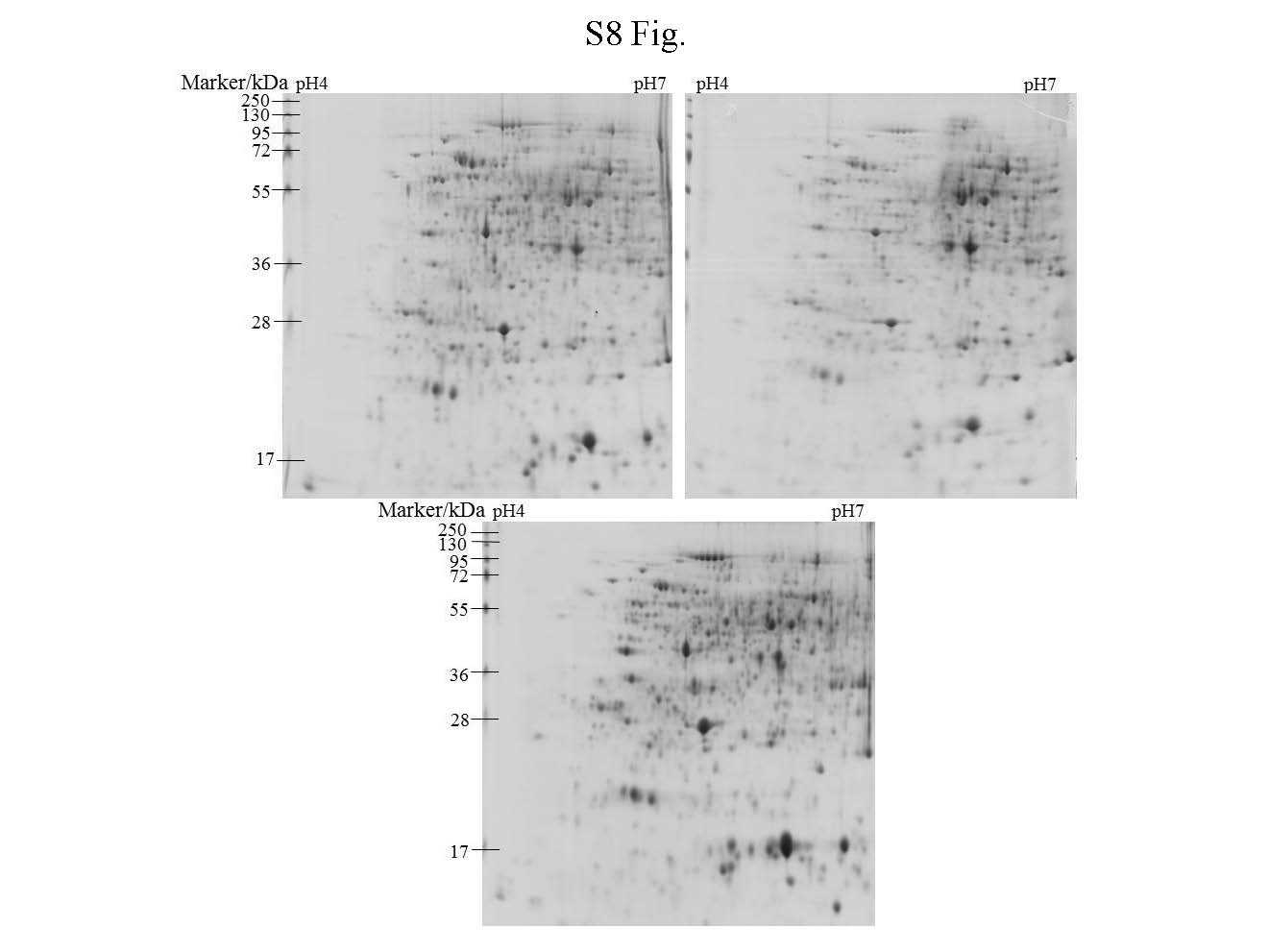

Supplement: S8 Fig — (TIF) [file pone.0174238.s011.tif]

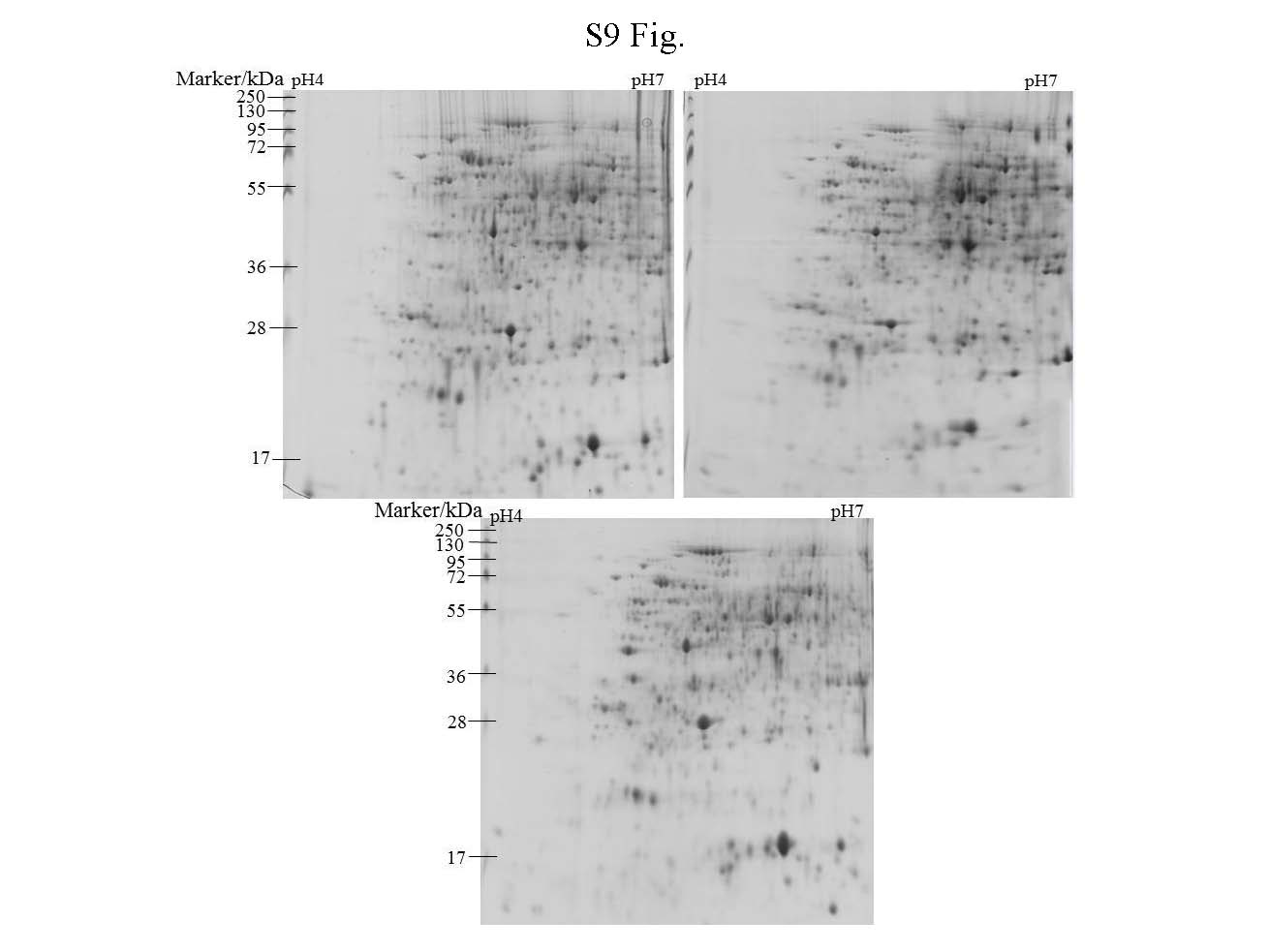

Supplement: S9 Fig — (TIF) [file pone.0174238.s012.tif]

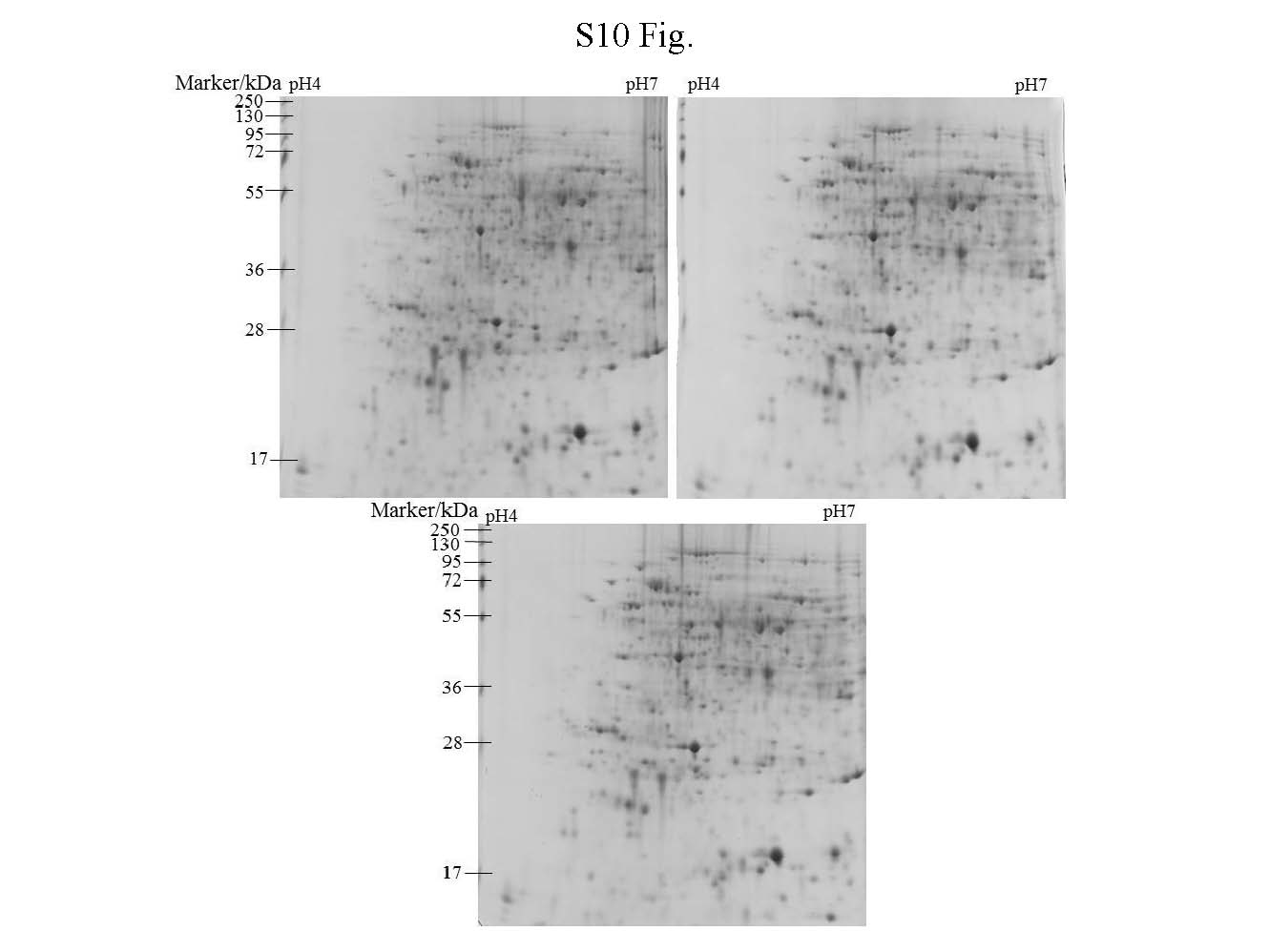

Supplement: S10 Fig — (TIF) [file pone.0174238.s013.tif]
